# Information transmission and processing in G-protein-coupled-receptor complexes

**DOI:** 10.1016/j.biosystems.2026.105706

**Published:** 2026-01-16

**Authors:** Roger D. Jones, Achille Giacometti, Alan M. Jones

**Affiliations:** a Department of Biology, University of North Carolina at Chapel Hill, Chapel Hill, NC 27514, USA; b Dipartimento di Scienze Molecolari e Nanosistemi, Università Ca’ Foscari Venezia, 30123 Venezia, Italy; c European Centre for Living Technology (ECLT) Ca’ Bottacin, 3911 Dorsoduro Calle Crosera, 30123 Venezia, Italy; d Department of Pharmacology, University of North Carolina at Chapel Hill, Chapel Hill, NC 27514, USA

**Keywords:** G protein-coupled receptor (GPCR), Information flow, Entropy, Second law of thermodynamics, Phosphatase, Nonequilibrium steady state, Induced-fit conformation, Drug design

## Abstract

We present a general theoretical framework for molecular computation in biological systems and apply it to G-protein-coupled receptors (GPCRs), which serve as central regulators of cellular information processing. Despite their importance, the physical principles underlying GPCR switching remain incompletely understood. Using nonequilibrium thermodynamics, we construct a model that identifies the parameters governing receptor-state transitions.

The framework shows that the configuration of the switch is governed by two factors: the ATP/GTP-driven chemical flux through the receptor complex and the free-energy difference between competing switch states. The model predicts that GPCRs can occupy three quasistable configurations, corresponding to “on”, “off”, and an intermediate state, each representing a local maximum in information transmission. Switch states can also be characterized by whether the switch supports a net chemical flux. Active states support sustained chemical flux, whereas inactive states do not.

The model incorporates reciprocal conformation-fit changes between ligand and receptor. As such, the model predicts that phosphatase activity, represented as an effective energy barrier, primarily determines whether the switch occupies the “on” or “off” state, whereas kinase activity maintains flux without directly setting state occupancy. These predictions based on experimental evidence point to new targets for drug design. Comparison with label-free impedance measurements supports the existence of multiple quasistable states that depend on ligand conformation.

Because the framework relies on general nonequilibrium principles rather than system-specific biochemistry, it extends naturally to other biological switching systems driven by chemical flux.

## 1. Introduction

Biological systems display complex behaviors that arise from interactions across multiple levels of organization. Although this complexity has led some to question whether biology can be described by a compact set of theoretical principles (Forestiero, 2022); concepts from thermodynamics, homeostasis (Bechtel and Bich, 2024), information theory (Bajić, 2024), and complexity science (Hébert-Dufresne et al., 2024) promise, however, to provide useful organizing frameworks. The purpose of this study is to develop a first-principles description of molecular computation that draws on these organizing ideas and to apply it to the important example of G protein coupled receptors (GPCRs), a major class of signaling proteins with central roles in physiology and pharmacology (Zhang et al., 2024; Koenig et al., 2017; Kosorok and Laber, 2019; Ginsburg and Phillips, 2018). Ultimately, the goal for such a model is to identify drug targets in order to improve health outcomes of drug therapies.

Cells process environmental information through networks of chemical reactions that act as molecular switches (Qian, 2007; Latorraca et al., 2018). These switches operate far from thermo-chemical equilibrium and require continuous input of energy. GPCRs are a clear example since their activity depends on GTP hydrolysis in G proteins and ATP driven phosphorylation and dephosphorylation cycles on the receptor. Without these fluxes, GPCR signaling would relax to equilibrium and lose functional capacity. This energy dependence suggests that biological computation may be shaped by evolutionary pressures that favor efficient transmission of information under physical constraints.

Recent experimental work highlights the need for theoretical models that account for these nonequilibrium features. Assays have begun to probe GPCR dynamics using photoisomerizable ligands and label-free measurements of cellular responses (Wirth et al., 2023). In this study, we analyze such measurements within a nonequilibrium thermodynamic framework that identifies two key control parameters: the chemical flux through the relevant reaction cycles and the free-energy difference between competing switch states. These parameters inform the ligand–receptor configuration and the amount of information that can be transmitted across the membrane. We also have theoretical indications that an important control parameter for switching is the height of the energy barrier that regulates flux from the on state to the off state.

The model predicts that GPCR switches can occupy up to three quasistable states, an on state, an off state, and an intermediate state, rather than the two states assumed in most biochemical models. The switch state depends on both the fixed chemical structure of the ligand and its inducible conformational changes. Experiments using photoactive ligands support this interpretation.

The theoretical approach is based on statistical mechanics and constrained optimization (Jaynes, 1957a,b) and extends previous work that applied information theory to biochemical networks (Jones and Jones, 2023a,b; Jones, 2024). It also generalizes standard mass action and Markov chain formulations, which assume constant reaction rates. This assumption does not hold for systems embedded within protein matrices that are continually modified by ligand binding and by ATP driven entropy flow. Under such conditions, reaction rates depend on the nonequilibrium environment, and the quasistable solutions appear in the theory.

Because switching behavior of this type also occurs in systems that lack GPCRs, for example, in plant signaling pathways, it is important to validate the theory in both general settings and in specific GPCR contexts. We therefore begin with label-free assays that report physical cellular changes without assuming a particular downstream mechanism. A precursor to this theory has already received experimental support, including in studies of biased signaling. Here, we replace that earlier framework on a stronger theoretical foundation and identify additional control parameters relevant to downstream signaling. Further development will involve extending the model to networks of interacting switches.

This paper is structured so that the main text presents general ideas, applications, and key theoretical results. The detailed mathematical development is provided in the Appendix, where a more complete description of the theoretical framework is given.

## 2. Methods

### 2.1. Schematic of the experiment

**Fig. 1.**
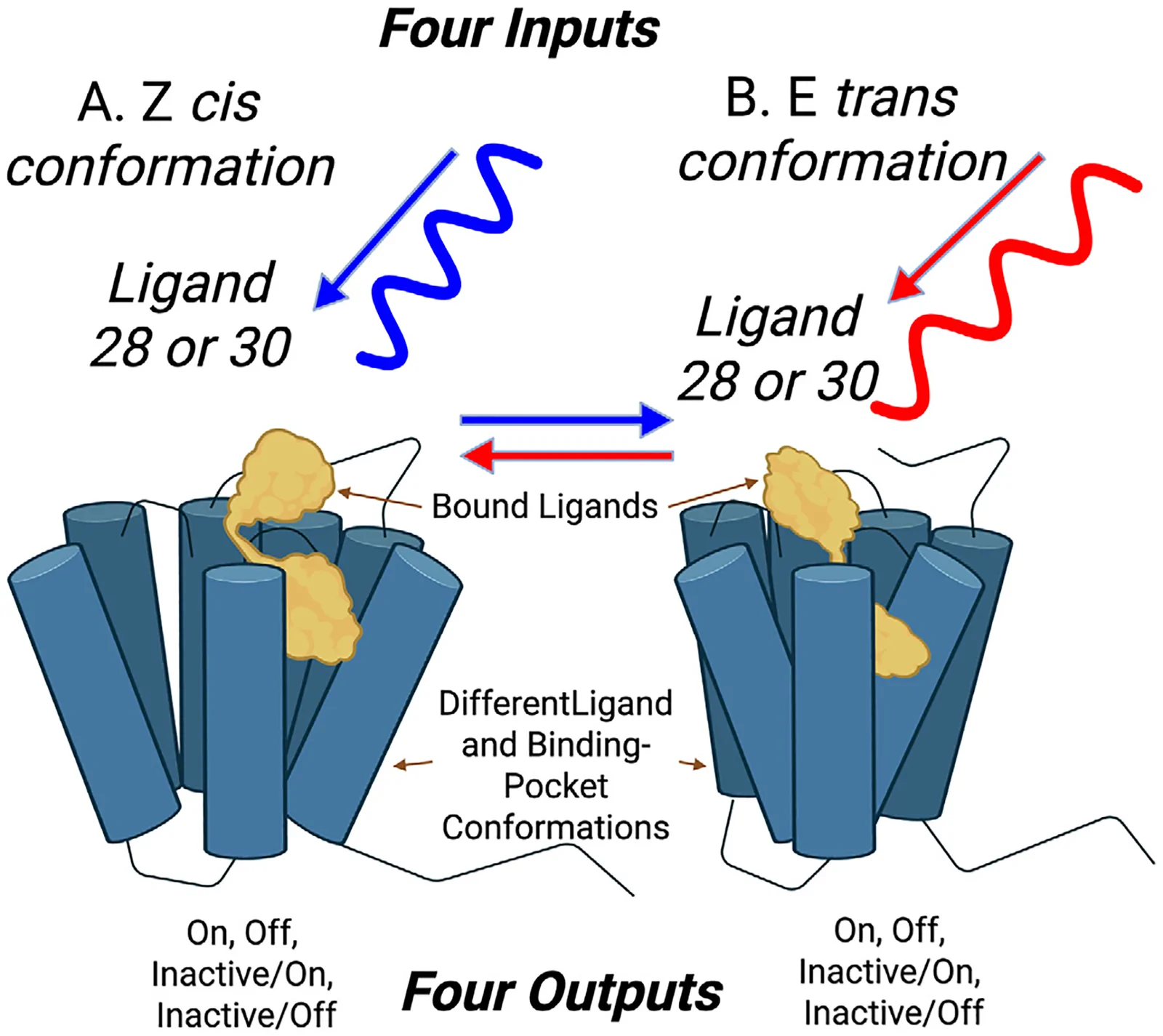
Conceptual schematic of the experiment. Ligands in cis (Z) or trans (E) configurations bind to GPCRs, while electrical impedance across a cell layer monitors signaling activity. Two engineered ligands (28 and 30) reversibly switch between Z and E isomers under wavelength-specific light (blue: Z→E; red: E→Z). Each conformation induces a distinct GPCR state, yielding four input combinations, encoding up to two bits of information.

Conceptual schematic of the experimental setup from Wirth et al. (2023) is shown in Fig. 1. Chinese hamster ovary cells (CHO) were genetically engineered to overexpress the receptor, a member of the neuropeptide Y family, and cultured to confluence on a gold foil substrate (Cabrele and Beck-Sickinger, 2000). This uniform expression of the receptor enabled for consistent detection of ligand-induced conformational changes via electronic impedance measurements across the gold foil.

Two wavelength-driven ligand isomers, cis (Z) and trans (E), were derived from azobenzene (ligand 28) and arylazopyrazole (ligand 30), producing four distinct GPCR inputs: E-28, Z-28, E-30, and Z-30. Z isomers were generated by 340 nm irradiation, while E-isomers were produced using 455 nm (ligand 28) and 528 nm (ligand 30) light. GPCR response was inferred from changes in cell morphology, monitored by impedance measurements (Skiba et al., 2022; Stolwijk et al., 2019; Wegener et al., 1999).

Natural downstream responses to activation of the $Y_{4}$ NPY receptor include reduced cAMP levels, context-dependent activation of PLC, and engagement of MAPK signaling pathways. The impedance measurement can also be viewed as a downstream, label-free response. In this interpretation, the switch behaves as a single conglomerate system that reflects the combined activity of the GTPase switch in the G protein and the phosphorylation–dephosphorylation cycles that generate the barcode.

No published data currently demonstrate that the specific receptor studied, when bound to the azobenzene- or arylazopyrazole-based ligands used in Wirth et al. activates a G protein and subsequently undergoes phosphorylation. Nevertheless, several recent studies have established that photoswitchable ligands can modulate GPCR activity in other systems. For example, Agnetta et al. showed that an azobenzenecontaining dualsteric ligand can reversibly alter receptor efficacy upon light stimulation (Agnetta et al., 2017). More recently, Duran-Corbera et al. demonstrated robust and reversible optical control of a native class A GPCR, the $\beta_{1}$ -adrenoceptor, using the photoswitchable ligand pAzo-2; light-induced *cis–trans* isomerization produced measurable changes in receptor activation in functional assays (Duran-Corbera et al., 2022). These studies collectively support the broader plausibility of using photoswitchable ligands to modulate GPCR signaling, even though direct evidence for the full sequence of events, ligand binding, G-protein activation, and phosphorylation, has not yet been reported for NPY with the specific ligands used here. Moreover, impedance measurements are a valid and measurable downstream response, even if not directly pharmacologically relevant. This is well demonstrated by Wirth et al. (2023).

#### 2.1.1. Non-mathematical schematic of the theory

The mathematical details of the framework are presented in Appendix A. Here we provide a conceptual outline of the approach without relying on formal derivations.

Biological information processing requires molecular systems to operate persistently far from equilibrium. Cells maintain these non-equilibrium steady states through continuous energy flow from nucleotide hydrolysis, such as ATP or GTP consumption, with the resulting entropy dissipated into the surrounding thermal environment. These steady states are analogous to biological homeostasis, since they reflect stable yet energy-driven configurations.

The theoretical framework begins with Landauer’s Principle, which states that information is associated with a thermodynamic cost and is linked to heat and entropy production (Sagawa, 2018). When a molecular switch erases information, it dissipates heat into the thermal bath (Jones et al., 2025b). Because energy is conserved, creating or transmitting information requires work. In biochemical systems this work is provided by conformational changes in the protein matrix in which the switch resides. For GPCR signaling, this matrix includes the receptor and its associated G proteins. The GPCR–G protein complex acquires usable energy from the excess concentration of extracellular ligand, and the binding reaction that includes ligand, receptor, and G protein is a ternary process (Burger et al., 2024).

The goal of the theory is to characterize the non-equilibrium steady states rather than the transient dynamics that lead to them. At steady state, the rate of information change is zero, which implies that the system occupies a local or global extremum of information flow. We assume that natural selection favors molecular organizations that maximize information flow under appropriate physical constraints. These constraints include the number of switches, the average energy of the switch configurations, and the average energy and entropy flux per switch.

Within this framework, optimization yields the fraction of time that each switch resides in inactive or active configurations, which correspond to states with zero or nonzero chemical flux, respectively. The theory also determines whether an active configuration is more likely to occupy the on or off state. These quantities provide the basis for predicting downstream cellular responses to ligand stimulation.

Previous theories do not incorporate information flow or account for the influence of natural selection on information-processing capacity. They also do not consider how the surrounding protein matrix modulates the magnitude of energy differences or chemical flux through molecular switches. The most established framework, the operational model (Black and Leff, 1983), restricts the system to only on and off states, which eliminates the possibility of a third quasistable configuration. In addition, earlier models do not include, or do not clearly describe, the roles of chemical flux, heat dissipation, entropy flow, or the explicit dependence on energy differences within the switch.

### 2.2. Application to G protein-coupled receptor complexes

To demonstrate and validate the proposed theoretical framework, we focus on the fundamental process of information processing on the cell membrane. Among such processes, signaling through GPCRs plays a central role, representing one of the most critical mechanisms in both biological regulation and medical intervention.

**Fig. 2.**
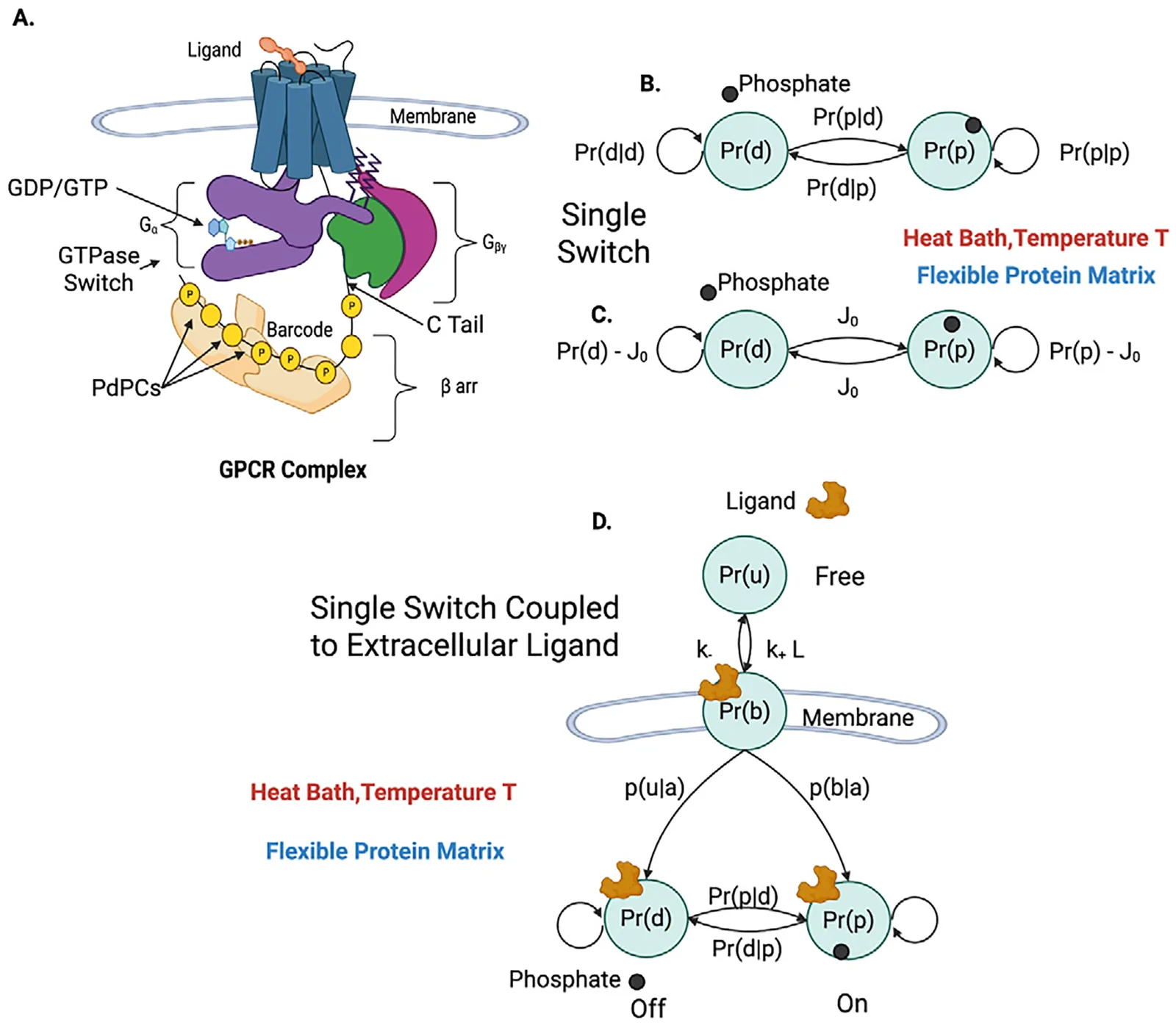
Simple Molecular Switch **A.** The 7-transmembrane GPCR is illustrated with blue cylinders representing the seven α helices that span the cell membrane. The extracellular ligand (orange) binds to the binding site of the GPCR inducing movement with the α helical bundles. The helices alter the conformation of the intracellular loops (thin black lines) of the GPCR complex. The intracellular portion of the complex has been separated for visibility. Two pathways may be activated, the $G_{\alpha }$ pathway (purple) and the βarr pathway (tan). The $G_{\alpha }$ subunit is a part of the G protein also composed of subunits β and γ. The βarr pathway is composed of additional response pathways determined by phosphorylation sites on the C tail of the GPCR and intracellular loops that form a barcode that encodes signals for downstream processes. **B.** Picture of a single switch taken to be a PdPC. A GTPase switch operates in the same manner. The switch resides in a heat bath at temperature T. In addition to the thermal bath, the switch is a component of a flexible protein complex that can modify energy barriers in the switch. Here, Pr(d) and Pr(p) are the probabilities of finding the receptor to be dephosphorylated and phosphorylated, respectively, while Pr(p∣d) and Pr(d∣p) are the conditional probabilities of finding the receptor has transitioned between phosphorylated to dephosphorylated positions and *vice versa*. **C.** Here, $J_{0}=\text{Pr}\left(p|d\right)\;\text{Pr}\left(d\right)=\text{Pr}\left(d|p\right)\;\text{Pr}(p)$ is the steady-state probability flux among states. The flux is kept finite due to an external energy/entropy source. **D.** Three-state model: free receptor (f), phosphorylated (p), and dephosphorylated (d). The bound state b is bound to the ligand but not the phosphate. The associated state b has two internal states d and p. These two states form the $G_{\alpha }$ switch. Ligand dissociation and association reaction rates are given by $k_{-}$ and $k_{+}$, respectively, and L is the ligand concentration. The picture for a GTPase switch is similar. A G protein-GDP is bound to Pr(a) and the ligand through a ternary reaction. The on state occurs when GDP is replaced with GTP.

The foundation of the current GPCR model is illustrated in Fig. 2, which schematizes the GPCR complex. In Fig. 2A, the 7-transmembrane GPCR (blue) spans the membrane, interacting with both extracellular ligands (orange) and intracellular G proteins (purple, green, violet).

Ligand binding induces a conformational change in the GPCR, which enhances its affinity for heterotrimeric G proteins. This results in the formation of a ternary complex, comprising the ligand, GPCR, and G protein—that is more stable than the ligand receptor complex alone (Mahoney and Sunahara, 2016).

The G protein α subunit $\left(G_{\alpha }\right)$ initially binds to GDP, representing the off state of the GTPase switch (Qian, 2007). Upon GPCR activation, GDP is released and replaced by GTP, whose cellular concentration is maintained far from equilibrium. This GTP binding activates $G_{\alpha }$, which completes the switch to the “on” state. The energy driving this transition derives from the thermodynamic disequilibrium between GDP and GTP.

The C-terminal tail of GPCR and the intracellular loops contain serine and threonine amino acid sites that can be phosphorylated/dephosphorylated by specific kinases/phosphatases. The C-terminal tail of GPCR and the intracellular loops contain serine and threonine amino acid sites that can be phosphorylated/dephosphorylated by specific kinases/phosphatases. The phosphorylation and dephosphorylation of these sites is known as a phosphorylation/dephosphorylation cycle (PdPC) that also acts as a molecular switch (Qian, 2007). Similarly to the GTPase switch, the “on” and “off” states of these PdPC switches are determined by the phosphorylation status of specific sites. However, in this case, the switches are powered by the high concentration of adenosine triphosphate (ATP), which drives the process (Berg et al., 2002).

The collection of these PdPCs establishes a distinct molecular pattern, called a barcode, which serves as a guide for downstream signaling processes (Yang et al., 2017; Chen et al., 2022). Adapters read this phosphorylation barcode to propagate a certain response (Fig. 2). In animals, this adapter is called β-arrestin (Latorraca et al., 2020) and in plants it is called VPS26A/B (Jones, 2023).

Furthermore, the flexible protein matrix surrounding the GPCR complex provides a mechanism to adjust energy levels and transition probabilities, facilitating changes in the conformation and behavior of the complex.

## 3. Results

### 3.1. Experimental results relevant to this study

If the four input conditions produce four independent output responses, this corresponds to two bits (log_2_ 4) of information transmitted across the membrane. Fewer distinct outputs would indicate reduced information transfer. If only the chemical structure of the ligand (for example, 28 versus 30) influences the response, then only one bit of information is transmitted. Observation of more than two outputs therefore implies that both the fixed chemical structure and the induced-fit conformations of the ligand (Franco et al., 2021) contribute to signaling.

The experiment reported in Wirth et al. (2023) revealed four distinct responses, demonstrating that both the Z and E photoisomerized conformations, along with the fixed structural differences between ligands 28 and 30, play essential roles in determining receptor output. This result indicates that each ligand can exist in two functionally distinct conformational states after light activation, enabling the full transmission of two bits of information through the receptor complex. These findings are consistent with independent observations of GTPase activity in other G proteins that display similar two-bit information transmission (Keshelava et al., 2018).

The ligand conformation was found to switch reversibly between *cis* and *trans* forms even while the ligand remained bound to the receptor. Importantly, removal of extracellular ligand did not alter the downstream response, which indicates that signaling is determined by the conformation of the bound ligand rather than by its continued presence in solution.

### 3.2. Theoretical results

The theory developed here generalizes the widely used operational model of Black and Leff (1983). In the operational model, the switch state is governed by multiple applications of Michaelis–Menten kinetics (Berg Jeremy et al., 2019), which produces a single solution for the output-state probability. In contrast, the framework presented in this study allows for up to three solutions, providing a richer and more flexible description of switch behavior.

The model described in Sections Appendix A.1, A.2 shows that a molecular switch can occupy quasistable states determined by two parameters: the chemical flux within the GPCR complex and the change of free energy of the switch. Both parameters are influenced by the characteristics of the extracellular ligand, its fixed and inducible conformations, and its concentration outside the cell.

This framework leads to the Biological Ensemble, a generalization of the Canonical Ensemble from equilibrium statistical mechanics (Reif, 2009). The Canonical Ensemble, described by Feynman as the “summit of statistical mechanics”^1^ (Feynman, 2018, Chapter 1), admits a single solution proportional to the exponential of the system energy (Reif, 2009). In contrast, the Biological Ensemble introduced here yields three possible solutions that turns the ensemble into a system of switches, a key result of this work.

We illustrate the power of the Biological Ensemble with an intuitive analogy. Near thermochemical equilibrium, a system relaxes to a single state, much as air molecules uniformly fill a room at fixed temperature and density. In our framework, if those molecules were in an NESS, the room could instead exist in one of three distinct states: one resembling the usual equilibrium condition and two others in which all molecules cluster into two different corners of the room. In this analogy, the NESS behaves as a switch with three possible configurations. It is clear that a room in equilibrium and a room in NESS are fundamentally different, and each would exhibit markedly different behavior.

The solutions correspond to local maxima of information flow, quasi-stable states that can transition under perturbation. Two parameters govern which state the switch occupies: (1) the energies of the phosphorylated $\left(E_{p}\right)$ and dephosphorylated $\left(E_{d}\right)$ conformations, and (2) the chemical flux $J_{0}$ from the phosphorylation/dephosphorylation cycle. Together, these parameters determine whether the switch is in the “off”, “on” or intermediate state.

The energy parameter is given by the normalized change in the Gibbs free energy going from the “off” to “on” switch configuration.

(1) $$\beta \left(E_{p}-E_{d}\right)$$

where β is the inverse of the temperature of the heat bath (Boltzmann constant = 1). The Gibbs free energy is the valid form of free energy to use since the system is in a heat bath at temperature T=1/β and no external work is being applied to the system. The energies $E_{p}$ and $E_{d}$ are modified from their baseline measured values (≈ 7 kcal/mol) (Rosing and Slater, 1972) by the presence of the protein matrix of the GPCR complex. Modifications as high as 17 kcal/mol have been seen in the muscles of athletes (Wackerhage et al., 1998).

If $R_{p}$ and $R_{d}$ are the numbers of receptors in the phosphorylated (“on”) and dephosphorylated (“off”) switch configurations, then the probability of the receptor being in each of the switch configurations is

(2) $$\text{Pr}(p)=\frac{R_{p}}{R_{d}+R_{p}}$$

and

(3) $$\text{Pr}(d)=1-\text{Pr}(p)=\frac{R_{d}}{R_{d}+R_{p}}$$

for the phosphorylated and dephosphorylated configurations, respectively.

We define the chemical flux $J_{0}$ as the flow of probability from one switch configuration to the other.

(4) $$J_{0}=\text{Pr}(d)\;\text{Pr}(p|d)=\text{Pr}(p)\;\text{Pr}(d|p)$$

where Pr(p∣d) is the probability that the receptor state transitions from configuration d to p and Pr(d∣p) is the probability of transition in the opposite direction. The equality sign is a consequence of the steady-state requirement and is an expression of Bayes Theorem (Grover, 2012).

The terms in Eq. (4) are intuitive from a chemist’s perspective. The probability Pr(d) is proportional to the chemical concentration of the system in the dephosphorylated configuration, while Pr(p) is proportional to the concentration of the phosphorylated concentration. The transition probabilities Pr(p∣d) and Pr(d∣p) are the reaction rates.

An intuitive schematic of the information landscape is provided in C, illustrating how the system navigates between quasistable states. The figure represents a landscape in which the curves are ridges of maximum switch information. Three solutions exist for the probability Pr(p) that the switch is in the “on” configuration for each flux and change in free energy value. In the limit of small flux, the solutions are (1) Pr(p)=1,Pr(d)=1−Pr(p)=0 (blue), (2) Pr(d)=1−Pr(p)=1,Pr(p)=0 (red), and (3) the Canonical Ensemble (yellow). We define solution (3) as the thermodynamic branch, while solutions (1) and (2) are defined to be the kinetic branch.

For small values of flux $J_{0}$, the highest information path, the thermodynamic branch (yellow), for probability Pr(p) obeys the Canonical Ensemble. The thermodynamic branch steepens as the chemical flux $J_{0}$ increases. At $J_{0}=\phi_{c}\approx 0.182$, the information content of the kinetic branch becomes equal to the information of the thermodynamic branch. For $J_{c}>\phi_{c}\approx 0.182$, the information in the kinetic branch is greater than the information in the Thermodynamic Branch. This implies that for flux greater than $\phi_{c}$, the states in the kinetic b ranch are more stable than the states in the thermodynamic branch. The opposite is true for $J_{0}<\phi_{c}$.

At $J_{0}=1/4$, the thermodynamic branch is given by Pr(p)=0. For $J_{0}>1/4$, a discontinuity forms in the kinetic branch and the two solutions exchange roles as the change in free energy increases.

**Fig. 3.**
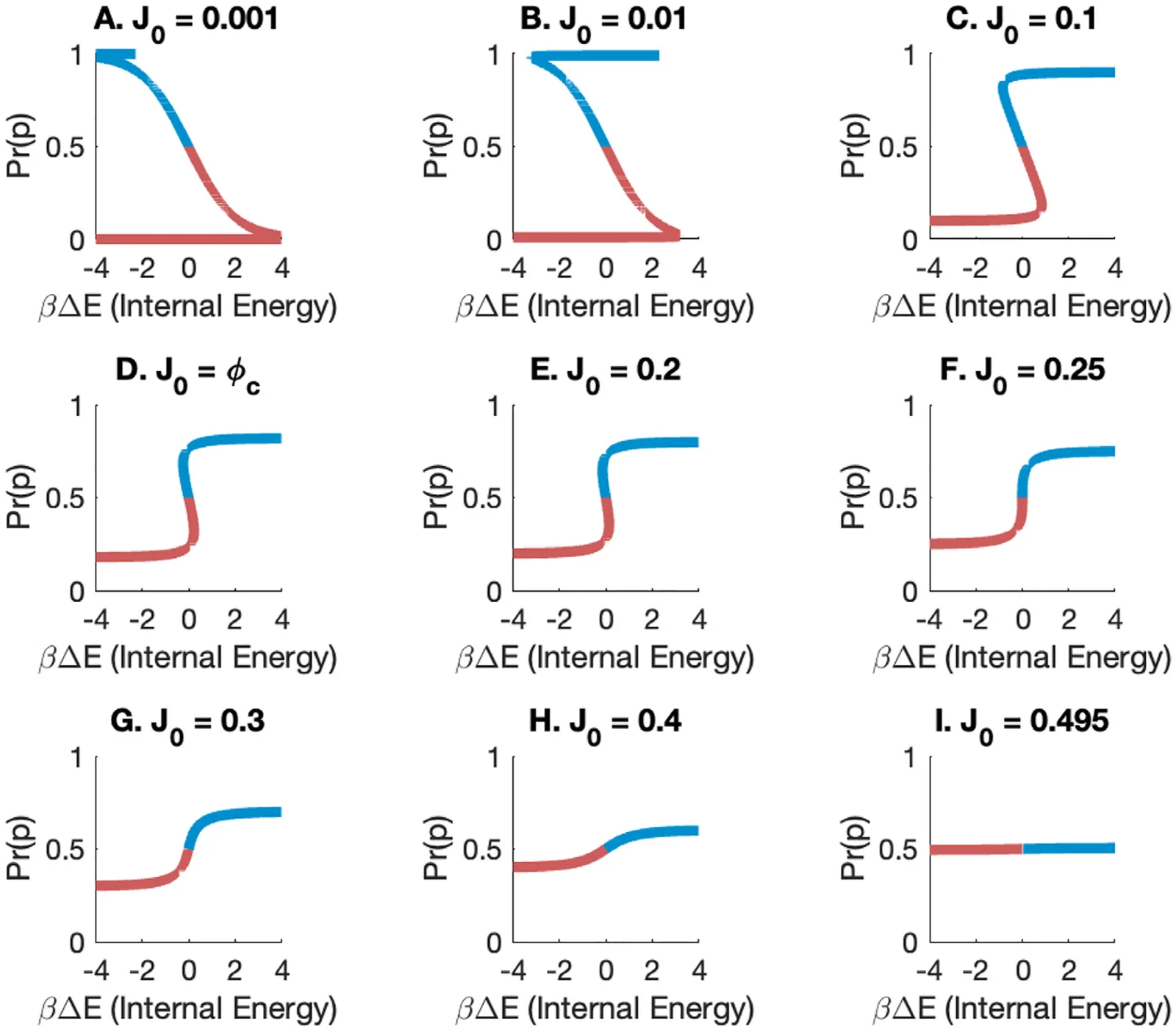
The quasistable solutions for Pr(p) for the Biological Ensemble as a function of the chemical flux $J_{0}$ and change in free energy between two receptor states $\beta \Delta E=\beta \left(E_{p}-E_{d}\right)$. **A.** In the limit of very small flux, the solutions are (1) Pr(p)=1,Pr(d)=1−Pr(p)=0 (blue), (2) Pr(d)=1−Pr(p)=1,Pr(p)=0 (red), and (3) the intermediate solution (0<Pr(p)<1). **B.-F.** The intermediate branch steepens as the chemical flux $J_{0}$ increases. **G.-I.** For high values of flux $J_{0}$, the intermediate branch becomes less steep. For fluxes less that the critical flux $\phi_{c}\approx 0.182$.

The curves in Fig. 3 represent local maxima of information transmission, corresponding to quasi-stable states defined by chemical flux $J_{0}$ and change in free energy $\beta \left(E_{p}-E_{d}\right)$. Here β is the inverse temperature of the heat bath, $E_{p}$ is the energy of the phosphorylated “on” state and $E_{d}$ is the energy of the dephosphorylated “off” state. These configurations act as local attractors, indicating preferred switch states within the control space.

Observed switch behavior is expected to align with these quasistable states. The Biological Ensemble framework shows how variations in $J_{0}$ and $\beta \left(E_{p}-E_{d}\right)$ guide the system into specific configurations, offering predictive insight into the switch dynamics under varying conditions.

A simple water-pump analogy, presented in the Discussion, offers a macroscopic visualization of the mechanism by which the switch transitions between these states.

**Fig. 4.**
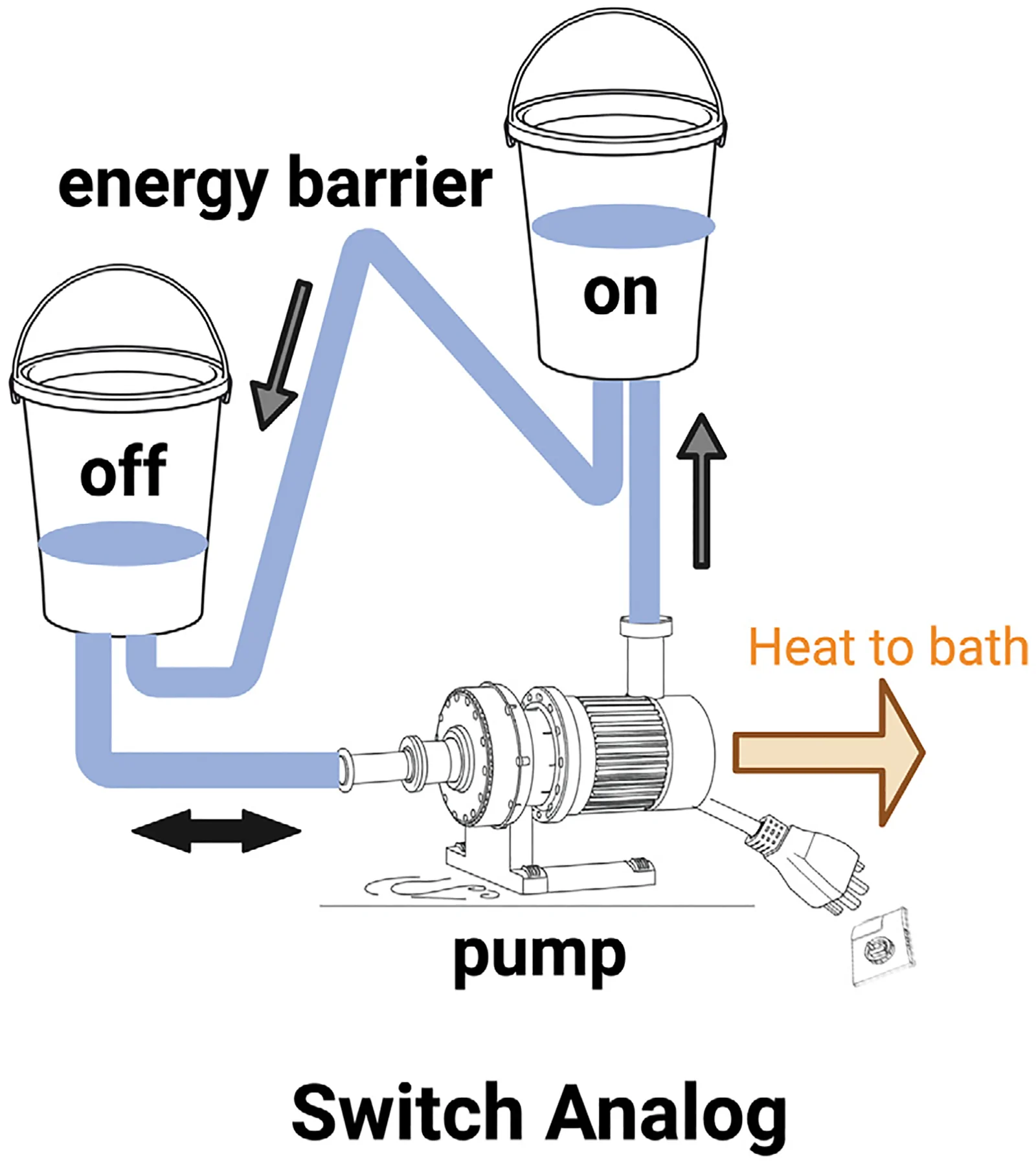
The “on” and “off” switch conformations are modeled as water buckets labeled on and off, where water levels represent the probability of each receptor state. Forward flow fills the on bucket, while reverse flow fills the off bucket. A pump, analogous to ATP/GTP-driven energy input, powers the cycle. The flow magnitude and state occupancy are regulated by an energy barrier, represented by the height of the return tube, which controls resistance to reverse flow.

A conceptual analogy (Jones et al., 2025a) is provided in Fig. 4. A pump drives water between two buckets that represent the on and off receptor states, while water height reflects state probability. The pump, analogous to an ATP or GTP driven kinase, maintains chemical flux. The energy barrier, represented by the return tube height, models phosphatase activity that regulates resistance to reverse flow. Heat generated by the pump dissipates into the surrounding environment. In this analogy, the phosphatase determines relative occupancy of the on and off states, whereas the kinase maintains flux.

The macroscopic hydraulic analog has three solutions similar to the three solutions in the mesoscopic model in Fig. 3.

### 3.3. Comparison of theory and experimental results

**Fig. 5.**
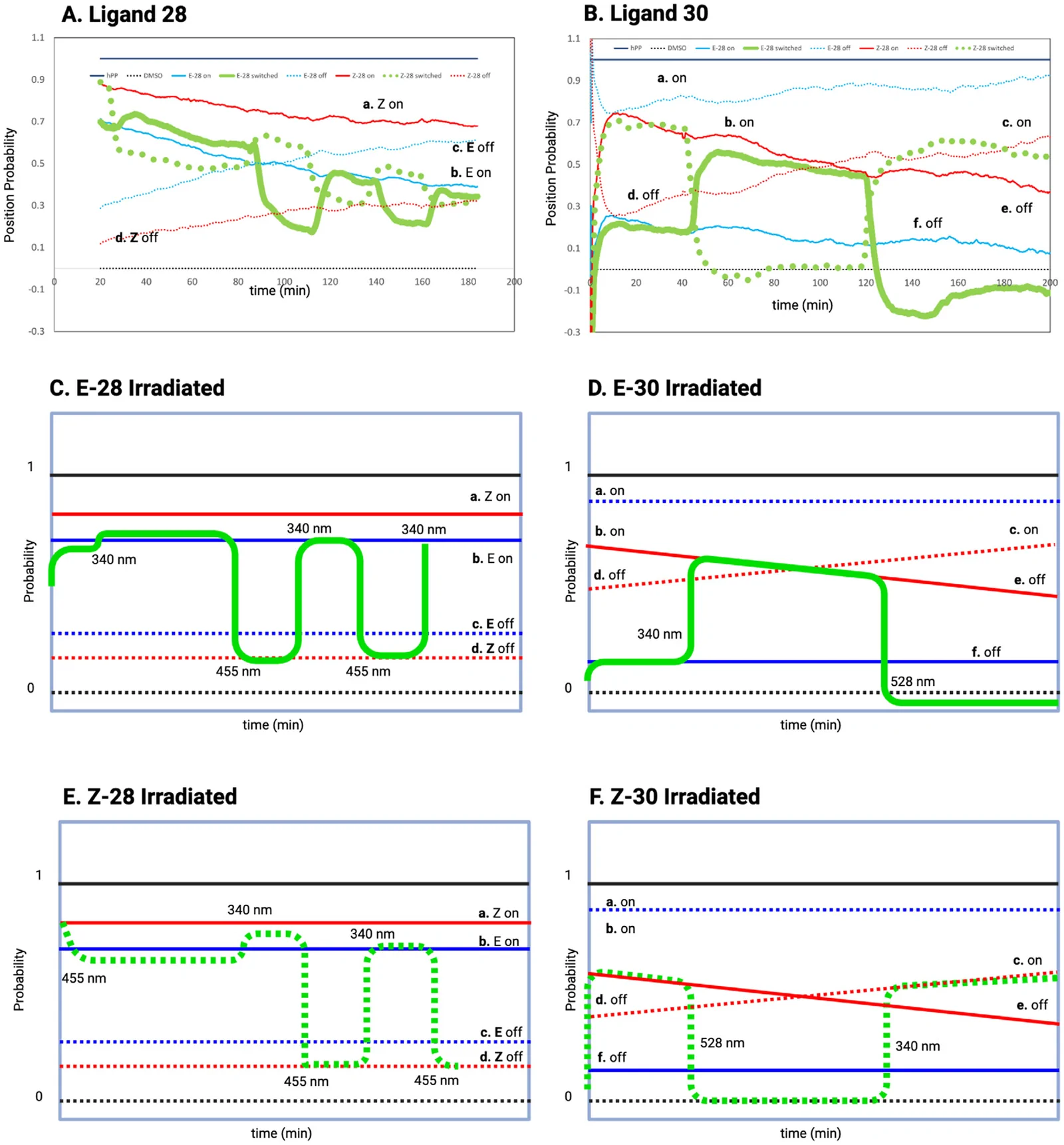
**A. and B.** Experimental Results **C.-F.** Schematic of Quasistable States. For Ligand 28, irradiation with wavelengths 340 nm and 455 nm toggle the system between the E/on (trans) and the Z/off (cis) states. For Ligand 30, irradiation with 340 nm and 528 nm toggles between active and inactive configurations.

The experimental results from Wirth et al. are depicted in Figs. 5A and B, alongside the theoretical predictions. The mapping from impedance to probability is given in B. Solid and dashed red lines show impedance responses for ligands 28 and 30 initially prepared in the Z/on and Z/off configurations, respectively; blue lines represent E/on (solid) and E/off (dashed) preparations. The solid black line shows the response to the endogenous ligand hPP, and the dashed black line corresponds to the solvent control (DMSO). Fig. 5A shows the results for ligand 28, and Fig. 5B for ligand 30. Green lines indicate ligands prepared in the Z/on (dashed) and E/on (solid) states that were alternately irradiated with short (340 nm) and long (455 nm for 28; 528 nm for 30) wavelengths.

Fig. 5C–F present schematic summaries of the experimental transitions among quasi-stable switch configurations. Fig. 5C shows that for ligand 28 in the E/on configuration, the impedance remains similar to that of Z/on. The off” states (E/off and Z/off) also overlap. Initial 340 nm irradiation slightly increases impedance but does not shift the system out of E/on. Subsequent alternating irradiation causes the system to toggle between E/on and Z/off, indicating that cis (Z) favors the “on” state and trans (E) favors “off”.

Fig. 5E shows the system initialized in the Z/on state. After a brief stable period, alternating light induces switching between Z/off and E/on, again confirming the correlation between the cis/trans states and switching on/off for ligand 28.

For ligand 30 (Figs. 5D and F), the Z/on and Z/off responses overlap, indicating that the “on”/”off” distinction does not apply. Instead, the system transitions between an active Z state that supports chemical flux and an inactive E state that does not. Thus, ligand 30 modulates switch activity versus inactivity, rather than discrete “on”/”off” signaling. Ligand 28 toggles the switch between on and off based on Z/E conformation, while ligand 30 toggles between active and inactive states. These behaviors reflect different modes of ligand control over GPCR switching.

**Fig. 6.**
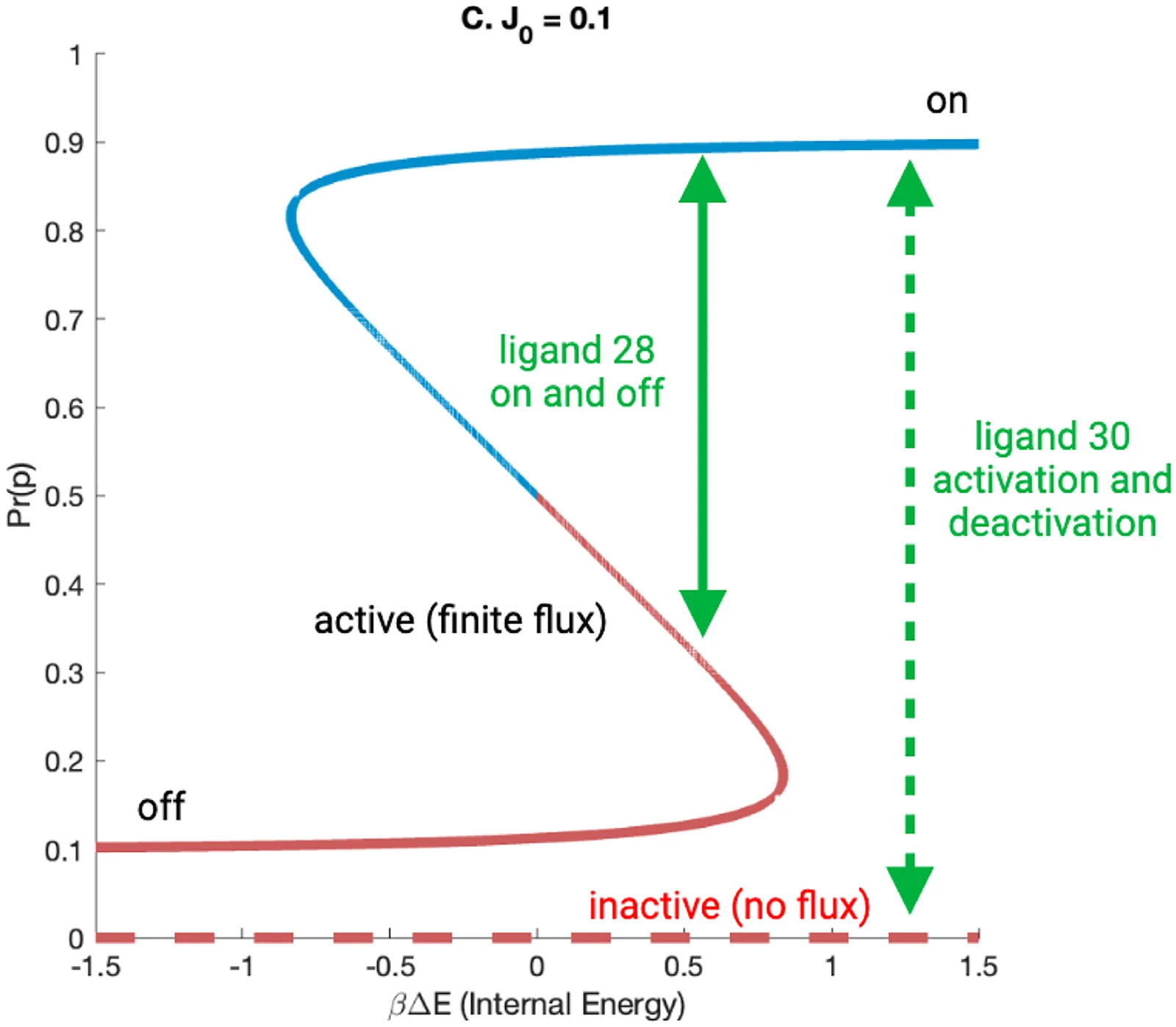
Enlargement of Figs. 3E and 3A. The solid blue and red curves are the active on (Pr(p)≥1/2) and active off (Pr(p)<1/2) states of the switch for $J_{0}=0.1$. The dashed red curve is the off state for an inactive switch $\left(J_{0}\to 0\right)$. The theory predicts that ligand 28 in the Wirth experiment toggles between on and off states, depending on the ligand conformation, while ligand 30 toggles between the active on state and the inactive state of the switch.

The mapping of the experimental observations onto the theoretical framework is shown in Fig. 6, which enlarges the regions highlighted in Figs. 3 E and A. The theory assumes that the system preferentially occupies active portions of the solution curves that support high information flow. The inactive state, corresponding to zero phosphorylation, appears as a dashed red curve in Fig. 6. Ligand 28 alternates between active on and active off states depending on its conformation, whereas ligand 30 toggles between an active on state and an inactive off state. In Fig. 6, the flux was set to $J_{0}=0.1$. When $\beta \left(E_{p}-E_{d}\right)$ is much less than one, thermal fluctuations destabilize the quasistable states. When it is much greater than one, the states become so stable that switching is no longer feasible, preventing computation. This tradeoff was already recognized by Schrödinger in early discussions of biological information flow (Schrodinger et al., 1992; Phillips, 2021). The Wirth experiment therefore implies that the flux $J_{0}$ and the energy $\beta \left(E_{p}-E_{d}\right)$ fall near 0.1 and 1, respectively, illustrating how these otherwise inaccessible parameters may be inferred indirectly from experimental assays.

## 4. Discussion

Biological switches function within nonequilibrium environments where continuous energy flow is required to sustain activity. The results presented here illustrate how such switches occupy nonequilibrium steady states rather than the equilibrium states emphasized in classical thermodynamics. These steady states depend on both chemical flux and free-energy differences, and together they generate a richer set of molecular responses than can be obtained from equilibrium analysis alone. The model shows that molecular computation arises naturally from the balance among energy dissipation, information flow, and structural constraints imposed by the protein matrix in which the switch resides.

A central strength of the thermodynamic approach is its ability to identify system-level properties while avoiding the need for detailed microscopic descriptions. Many complex systems converge to characteristic final states that can be predicted from coarse variables. Molecular switches behave in the same way. Although the microscopic paths taken during signaling are highly heterogeneous, the steady states reached by the system depend primarily on the underlying energy landscape and on the fluxes that maintain the system away from equilibrium. This perspective aligns with Jaynes’s information-theoretic approach (Jaynes, 1957a), which seeks to identify the macroscopic constraints that shape system behavior.

In the context of GPCRs, chemical flux provides the driving force that maintains switching among intermediate, off, and on configurations. Information erasure within these molecular machines produces heat, and the associated entropy flow requires sustained input of ATP or GTP. To capture this behavior, we introduced the Biological Ensemble, which extends classical free-energy minimization to include constraints from chemical flux and information transmission. This formulation predicts switching dynamics that are not accessible through equilibrium models or through kinetic schemes that assume constant reaction rates.

A hydraulic analogy helps illustrate these principles. In this analogy, the pump represents the kinase activity that sustains chemical flux, and the barrier height represents phosphatase activity that regulates transitions between states. Increasing the barrier shifts occupancy toward the on state, while lowering it favors the off state. If the barrier becomes too large, flux cannot be maintained, which reflects the loss of function when molecular systems become trapped in high-energy configurations. This analogy highlights the distinct roles of kinase and phosphatase activities in determining both flux and state occupancy.

The theory predicts two broad regimes of behavior. Near equilibrium, at low flux, the system follows thermodynamic predictions dominated by thermal fluctuations. As flux increases, the system enters a kinetic regime characterized by nonlinear effects and the appearance of multiple quasistable states. These additional states represent local maxima in information transmission and cannot be captured by traditional mass-action or Markov-chain models that assume fixed reaction rates. The transition between regimes marks a qualitative shift in system organization and information-processing capacity.

Ligand structure and availability regulate both the free-energy difference between states and the flux that sustains switching. The impedance measurements analyzed here reveal four distinct outputs that correspond to combinations of active and inactive states and on and off configurations. Because the measurements are label-free, the assay does not distinguish between GTPase-driven and phosphorylation-driven mechanisms, yet the theoretical framework applies equally to both. The presence of four outputs indicates that ligand conformation and ligand identity contribute independent bits of information, consistent with two-bit transmission in other G-protein systems.

Although the present analysis focuses on GPCR signaling, the principles are broader. Binary reaction-based switches are widespread in biology, and nucleotide exchange and phosphorylation cycles function as universal motifs in regulatory pathways. Similar architectures govern switching in receptor tyrosine kinases, Toll-like receptors, and plant signaling systems. The present framework therefore provides a general thermodynamic basis for understanding biological information processing across diverse contexts.

The theory also identifies control parameters that are not considered in precursor models, including the barrier height that determines the ease of transition between states. Further controls are expected to emerge when the theory is extended to arrays of interacting switches, and this will be addressed in future work. The transition from inactive switch states (J=0) to active states (J>0) is subtle and requires the interaction of multiple switches. This is the topic of follow-up work. Additional experimental comparisons, particularly those that examine downstream branches of GPCR signaling, will be essential for evaluating the generality of the framework.

## 5. Conclusions

1. A general theoretical framework for molecular computation in biological systems has been developed using nonequilibrium thermodynamics and information theory.
2. The theory predicts that biochemical switches, including GPCR complexes, can occupy up to three quasistable states—on, off, and an intermediate state, each associated with a local maximum in information transmission.
3. Two fundamental control parameters determine switch behavior:
  - the nonequilibrium chemical flux generated by phosphorylation or GTPase cycles, and
  - the free-energy difference between the phosphorylated and dephosphorylated states
4. Mapping the theory to light-controlled impedance assays from Wirth et al. (2023) shows that the experimental responses are consistent with transitions among the predicted quasistable states.
5. The analysis suggests that phosphatase activity primarily sets on/off occupancy by controlling the effective energy barrier, while kinase activity sustains flux without directly selecting the switch configuration.
6. Because the framework relies on general nonequilibrium principles rather than GPCR-specific biochemistry, it has the potential to be broadly applied to other biological switching systems.

## Figures and Tables

**Fig. C.7.**
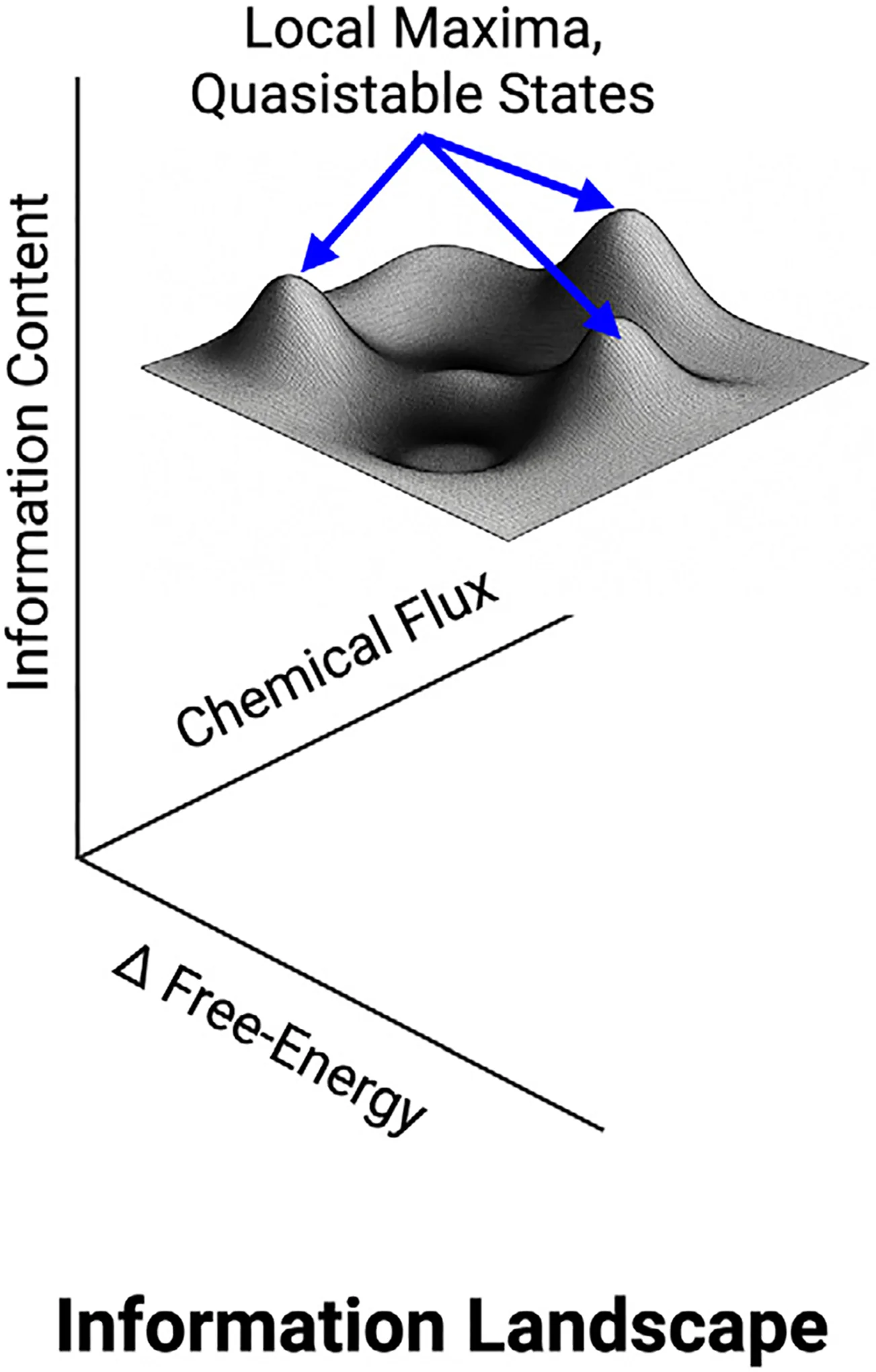
The two control parameters are given by the change in the free energy of phosphate bonding, $\beta \left(E_{p}-E_{d}\right)$, and the chemical flux, $J_{0}$. The information content of the system is displayed on the vertical axis. Local maxima and minima of the information are locations of steady state for information content of the system.

## Data Availability

The authors do not have permission to share data.

## Footnotes

1 “This fundamental law is the summit of statistical mechanics, and the entire subject is either the slide-down from this summit, as the principle is applied to various cases, or the climb-up to where the fundamental law is derived and the concepts of thermal equilibrium and temperature T clarified”. R. P. Feynman.

## Appendix A. Theory

### A.1. Information and the second law of thermodynamics

Standard equilibrium techniques that analyze systems by minimizing free energy do not apply to information-processing systems. Because information processing requires continuous energy and entropy flow, even in steady state (Landauer, 1996), entropy maximization is not achievable in non-isolated systems (Jones et al., 2025b).

The entropy S of system composed of W states x∈𝒳 is

(A.1)
$$S=-\sum_{i=1}^{W}\text{Pr}\left(x_{i}\right)\;\text{ln}\;\text{Pr}\left(x_{i}\right)$$

Assume that the system is composed of a switch in the form of a chemical reaction

(A.2)
d⇌p

where, as an example, d is the dephosphorylated state of the switch and p is the phosphorylated state of the switch. The rest of the system is a heat bath that absorbs heat that is generated by the chemical reaction. The entropy, Eq. (A.1), can then be written

(A.3)
$$S=S_{m}-I_{M}$$

where $S_{m}$ is the entropy of the microstates given that the observables d and p is the switch are independent and $I_{M}$ is the information in the correlation of the mesoscopic components d and p of the switch. The microscopic entropy $S_{m}$ is composed of two pieces, the entropy of the microscopic components of the switch and the microscopic entropy of the microstates of the external heat bath. The entropy of the microstates is

(A.4)
$$S_{m}=\sum_{i=1}^{W}\text{Pr}\left(x_{i},d,p\right)\;\text{ln}\left[\frac{\text{Pr}(d,p)}{\text{Pr}\left(x_{i}\right)\text{Pr}(d)\text{Pr}(p)}\right]$$

where Pr(d,p) is the joint probability of d and p.

The mutual information (Shannon and Weaver, 1963) associated with the correlation of d and p is

(A.5)
$$I_{M}=\sum_{\left\{d,p\right\}}\text{Pr}(d,p)\;\text{ln}\left[\frac{\text{Pr}(d,p)}{\text{Pr}(d)\text{Pr}(p)}\right]$$

The Second Law of Thermodynamics states that the entropy of the system increases with time or achieves a maximum value.

(A.6)
$$\Delta S=\Delta S_{m}-\Delta I_{M}\geq 0$$

If the number of states W is infinite, the system never achieves a maximum value.

In the case that the switch achieves a steady state $\Delta I_{M}\to 0$, then all the entropy generated by the switch goes into the heat bath.

(A.7)
$$\Delta S\to \Delta S_{m}\geq 0$$

When the switch has reached steady state, the switch information $I_{M}$ is at an extremum.

For a system with an infinite number of states, W→∞, total entropy continues to increase indefinitely. Local regions of correlation, such as molecular switches, can achieve steady states even as the entropy of the larger system grows. The heat generated by the switch is transferred to the surrounding heat bath. This scenario contrasts with finite systems W, where the total entropy S can reach a maximum. Because biological information processing systems function far from equilibrium, traditional frameworks based on minimizing Gibbs free energy G=E−TS, with E as the energy of the system and T the temperature of the bath do not apply.

### A.2. Theoretical model of the switch

Building on our earlier theoretical framework (Jones and Jones, 2023a,b; Jones, 2024), which successfully predicted the results of the experimental assay by maximizing the flow of information from the extracellular environment to the intracellular region of the GPCR complex, the present work extends this theory by incorporating a higher temporal resolution. This enhanced model is specifically developed to interpret the detailed, time-dependent dynamics observed in recent impedance-based experiments by Wirth et al. (2023). In line with its predecessor, this updated model is guided by the fundamental principle that natural selection favors molecular systems optimized to maximize information transmission capacity (Shannon and Weaver, 1963), thus providing a more granular and dynamic view of the GPCR switching mechanisms.

Figs. 2B and 2C presents a simplified mathematical representation of the schematic shown in Fig. 2A. This abstraction allows for a more tractable analysis using the tools of statistical mechanics (Reif, 2009). This level of simplification is common and has been useful in physics (Dönertaş, 2011; Ireson, 2005). In this model, the GPCR complex is reduced to a single switch embedded in a heat bath and surrounded by a flexible protein matrix (Karshikoff et al., 2015; Gerstein and Echols, 2004; Marsh et al., 2012; Jacobs et al., 2001; Sauer et al., 2020; Bernadó et al., 2007; Teilum et al., 2011, 2009; Homans, 2007). The probabilities Pr(d) and Pr(p) represent the fraction of time the receptor spends in the dephosphorylated and phosphorylated states, respectively. Specifically, the d position corresponds to the state in which the G protein is bound to a GDP molecule, while the p position corresponds to the state in which GDP has been phosphorylated to form GTP. During a time interval Δt, the switch can transition between these states with transition probabilities Pr(d∣p) and Pr(p∣d), or it can remain in the same state with probabilities Pr(d∣d) and Pr(p∣p). The heat bath ensures that the fluctuations between these switching states occur as if the system is at a temperature T=1/β, where T is in energy units.

Fig. 2C describes the switch in terms of chemical flux

(A.8)
$$J_{0}=\text{Pr}\left(p|d\right)\;\text{Pr}\left(d\right)=\text{Pr}\left(d|p\right)\;\text{Pr}(p)$$

which represents the flow of probability from one switch position to another. This description is useful if the switch is fueled by an external source such as a GTP/GDP disequilibrium. A flow of energy from GTP/GDP into the switch is turned into heat $Q_{⊙}$ in the time interval Δt

(A.9)
$$Q_{⊙}=J_{0}\;q=-\Delta G_{⊙}$$

where −q is the amount of heat released when a GPCR transitions from the “off” position to the “on” position and back; and ΔG is the amount of the free energy from GTP that is injected into the switch in time Δt. The minus sign indicates that the heat is leaving the switch. Eq. (A.9) describes the flow of entropy through the switch. A flow of entropy of this nature is essential for life (Morowitz and Smith, 2007). We take flux $J_{0}$ as the external driver of emergent behavior in the switching system.

The details of ligand/receptor binding are an important part of the overall picture. The inclusion of ligand kinetics is shown in Fig. 2D. The interaction between the ligand and the free receptor u to form the bound state b is modeled using Michaelis–Menten kinetics (Berg Jeremy et al., 2019). The forward reaction rate is given by $k_{+}L$ where L represents the ligand concentration, while the reverse reaction rate is $k_{-}$

Within the bound state b (Fig. 2), the receptor can adopt different internal states corresponding to the switch positions. The probability that the receptor is in the dephosphorylated “off-”state d given that it is in b is denoted as Pr(d∣b). Similarly, the probability that the receptor is in the phosphorylated “on” state is Pr(p∣a). The observed probabilities Pr(d) and Pr(p) are

(A.10)
$$\text{Pr}\left(d\right)=\text{Pr}\left(b\right)\;\text{Pr}(d|b)$$

and

(A.11)
$$\text{Pr}\left(p\right)=\text{Pr}\left(b\right)\;\text{Pr}(p|b)$$

Aspects of the ligand/receptor binding kinetics were observed in Wirth et al. (2023). Specifically, it was observed that the ligands remained bound to within the time range of the experiment even if the extracellular fluid was washed free of ligands. The details of these observations provide insight into the role that the switch position plays in ligand binding. In this study, we do not address this process. This will be addressed in a follow-up study.

The transmission of information I (Shannon and Weaver, 1963; Ash, 2012; Frigg and Werndl, 2011) through the switch is:

(A.12)
$$I=\sum_{i\in \{d,p\}}\sum_{j\in \{d,p\}}\text{Pr}\left(j|i\right)\;\text{Pr}\left(i\right)\;\text{ln}\left(\frac{\text{Pr}(i|j)}{\text{Pr}(i)}\right)$$

(A.13)
$$I=I\left(d,p\right)+\left(\text{Pr}\left(d\right)-J_{0}\right)\;\text{ln}\left(\frac{\text{Pr}\left(d|d\right)}{\text{Pr}\left(d\right)}\right)+\left(\text{Pr}\left(p\right)-J_{0}\right)\;\text{ln}\left(\frac{\text{Pr}(p|p)}{\text{Pr}(p)}\right)$$

(A.14)
$$I(d,p)=J_{0}\;\text{ln}\left(\frac{\text{Pr}\left(d|p\right)\;\text{Pr}(p|d)}{\text{Pr}\left(d\right)\;\text{Pr}(p)}\right)$$

Maximization of the information transmitted through the switch subject to the constraints that the average energy $\bar{E}$ of the switch is given and the flux $J_{0}$ is an externally determined constant leads to what we refer to as the Biological Ensemble

(A.15)
$$\beta \Delta E=\beta \left(E_{p}-E_{d}\right)=\text{ln}\left[\frac{Pr(d{)}^{2}\left[\text{Pr}(p)-J_{0}\right]}{\text{Pr}(p{)}^{2}\left[\text{Pr}(d)-J_{0}\right]}\right]-\frac{2\left(\text{Pr}(p)-J_{0}\right)}{\text{Pr}(p)}+\frac{2\left(\text{Pr}(d)-J_{0}\right)}{\text{Pr}(d)}-\frac{2J_{0}[1-2\;\text{Pr}(p)]}{\text{Pr}(p)\;\text{Pr}(d)}$$

(A.16)
$$\bar{E}=\text{Pr}(d)\;E_{d}+\text{Pr}(p)\;E_{p}$$

where $E_{d}$ is the energy of the switch in the off position and $E_{p}$ is the energy of the switch in the on position. The word “ensemble” is commonly used in two related concepts. It can be used to describe the probability given by Eq. (A.15) of finding a switch in the p position or it can describe an imaginary collection of switch states that have probability given by Eq. (A.15) or another equation such as the Canonical Ensemble (Reif, 2009).

The Biological Ensemble, Eq. (A.15), determines the theoretical outcomes and testable predictions that can be used to inform the experimental observations of Wirth et al. (2023). Like the Canonical Ensemble in equilibrium thermodynamics (Reif, 2009), the Biological Ensemble can predict microscopically observable behavior such as the impedance measurements described in the previous section. To see this, we assumed that the switch positions are correlated with the GPCR conformations generated by the Z and E forms of the ligand. It follows that Eq. (A.15) predicts the relationships among the impedance observations in Wirth et al. (2023)

The solutions in Fig. 3 represent the local maximums of information transmission. Two parameters determine the system output, the change in internal energy $\beta \Delta E=\beta \left(E_{p}-E_{d}\right)$ and the externally driven flux $J_{0}$. Rapid shifts in these two quantities may initiate a shift from one local maximum to another.

For most pharmaceutical applications, the control parameters are altered by changes in the ligand and ligand conformation. Of particular importance are jumps initiated by a change in ligand or ligand conformation that move the switch from the “on position, Pr(p)→1 to the “off position, Pr(d)=1−Pr(p)→1. One type of jump among solutions is particularly important, jumps in which changes in ligand conformation lead to changes in the switch position. This permits the ligand, which may be a drug, to control the downstream cellular response to the ligand. In this study, we focus on the identification of these jumps in the observations in Wirth et al. (2023). This informs the efficacy of a drug candidate.

## Appendix B. Connection between experiment and theory

The experimenters in Wirth et al. (2023) assumed the existence of a strong correlation between the measured impedance and the downstream response. We explicitly assume the simplest relationship between impedance and response, which is linear

(B.1)
$$\text{Pr}(d)=1-\text{Pr}(p)=\frac{Z-Z_{\text{DMSO}}}{Z_{hPP}-Z_{\text{DMSO}}},$$

where $Z_{hPP}$ is the impedance measured with the endogenous agonist human pancreatic polypeptide (hPP) and $Z_{\text{DMSO}}$ is the impedance for the solvent DMSO (negative control). The Biological Ensemble is symmetric upon interchange of p and d. Therefore, Eq. (B.1) with p replaced with d is also a possible connection between experiment and theory. As long as the relationship between Pr(p) and Z is monotonic, the conclusions of this paper should not be affected by the exact form of Eq. (B.1). hPP and DMSO were used to define the boundaries of observable impedance in Wirth et al. (2023). The connection between impedance Z and probability of the “on” position Pr(d) provides visibility into how flux $J_{0}$ and change in free energy $\beta \left(E_{p}-E_{d}\right)$ change in response to various ligand molecules and conformations of the molecules. Fig. 3 provides the map for the relationship between impedance and control parameters $J_{0}$ and change in free energy for each ligand.

## Appendix C. Information landscape

Fig. C.7 presents a conceptual schematic of the information landscape described in Section 3.2, with the two control parameters, $\beta \left(E_{p}-\right.E_{d})$ and $J_{0}$, plotted along the horizontal axes and the information content of the system on the vertical axis. The surface contains peaks and valleys, representing points where the time derivative of the information content is zero. These extrema correspond to the NESS of the system. The specific NESS for the modeled system are mapped in Fig. 3. Each ridge in Fig. C.7 marks a local information maximum, although not necessarily the global maximum.

The local maxima of the Biological Ensemble solutions are shown in Fig. 3. For a system residing in one of these local maxima to transition to another local maximum, it must experience a sufficiently large perturbation to overcome the stability of the local state.

At the critical flux

(C.1)
$$J_{0}=\phi_{c}\approx 0.182$$

The maximum information in the thermodynamic branch and the kinetic branch is equal at free energy $\beta \Delta E=\beta \left(E_{p}-E_{d}\right)$. As the flux $J_{0}$ increases, the regimes eventually merge into a single continuous solution at the maximum information value of $J_{0}=1/2$, as seen in Fig. 3I.

## Glossary

ATP: Adenosine Triphosphate
CHO: Chinese hamster ovary
GPCR: G Protein Coupled Receptor
GTP: Guanosine Triphosphate
NESS: Nonequilibrium Steady State

## References

[R1] Agnetta L, Kauk M, Canizal MCA, Messerer R, Holzgrabe U, Hoffmann C, Decker M, 2017. A photoswitchable dualsteric ligand controlling receptor efficacy. Angew. Chem. Int. Ed. 56 (25), 7282–7287. 10.1002/anie.201701524 28510314

[R2] Ash RB, 2012. Information Theory. Courier Corporation.

[R3] Bajić D, 2024. Information theory, living systems, and communication engineering. Entropy 26 (5), 430. 38785679 10.3390/e26050430 PMC11120474

[R4] Bechtel W, Bich L, 2024. Situating homeostasis in organisms: maintaining organization through time. J. Physiol. 602 (22), 6003–6020. 39383321 10.1113/JP286883

[R5] Berg JM, Tymoczko JL, Stryer L, , 2002. Biochemistry. WH Freeman, New York.

[R6] Berg Jeremy M, Tymoczko John L, Gatto Gregory J Jr., Lubert S, 2019. Biochemistry. W. H. Freeman.

[R7] Bernadó P, Mylonas E, Petoukhov MV, Blackledge M, Svergun DI, 2007. Structural characterization of flexible proteins using small-angle X-ray scattering. J. Am. Chem. Soc. 129 (17), 5656–5664. 17411046 10.1021/ja069124n

[R8] Black JW, Leff P, 1983. Operational models of pharmacological agonism. Proc. R. Soc. Lond. Ser. B. Biological Sci. 220 (1219), 141–162. 10.1098/rspb.1983.0093 6141562

[R9] Burger WA, Draper-Joyce CJ, Valant C, Christopoulos A, Thal DM, 2024. Positive allosteric modulation of a GPCR ternary complex. Sci. Adv. 10 (37), eadp7040. 10.1126/sciadv.adp7040 PMC11389776 39259792

[R10] Cabrele C, Beck-Sickinger AG, 2000. Molecular characterization of the ligand–receptor interaction of the neuropeptide Y family. J. Pept. Sci.: An Off. Publ. Eur. Pept. Soc. 6 (3), 97–122. 10.1002/(SICI)1099-1387(200003)6:3<97::AID-PSC236>3.0.CO;2-E 10759209

[R11] Chen H, Zhang S, Zhang X, Liu H, 2022. QR code model: a new possibility for GPCR phosphorylation recognition. Cell Commun. Signal. 20 (1), 1–16. 35236365 10.1186/s12964-022-00832-4 PMC8889771

[R12] Dönertaş Ş, 2011. Role of Thought Experiments in Solving Conceptual Physics Problems. Middle East Technical University.

[R13] Duran-Corbera A, Faria M, Ma Y, Prats E, Dias A, Catena J, Martinez KL, Raldua D, Llebaria A, Rovira X, 2022. A photoswitchable ligand targeting the β1-adrenoceptor enables light-control of the cardiac rhythm. Angew. Chem. Int. Ed. 61 (30), e202203449. 10.1002/anie.202203449 PMC9401038 35608051

[R14] Feynman RP, 2018. Statistical Mechanics: A Set of Lectures. CRC Press.

[R15] Forestiero S, 2022. The historical nature of biological complexity and the in-effectiveness of the mathematical approach to it. Theory Biosci. 141 (2), 213–231. 35583727 10.1007/s12064-022-00369-7 PMC9184406

[R16] Franco R, Rivas-Santisteban R, Reyes-Resina I, Navarro G, 2021. The old and new visions of biased agonism through the prism of adenosine receptor signaling and receptor/receptor and receptor/protein interactions. Front. Pharmacol. 11, 628601. 33584311 10.3389/fphar.2020.628601 PMC7878529

[R17] Frigg R, Werndl C, 2011. Entropy-a guide for the perplexed.

[R18] Gerstein M, Echols N, 2004. Exploring the range of protein flexibility, from a structural proteomics perspective. Curr. Opin. Chem. Biol. 8 (1), 14–19. 15036151 10.1016/j.cbpa.2003.12.006

[R19] Ginsburg GS, Phillips KA, 2018. Precision medicine: from science to value. Health Aff. 37 (5), 694–701. 10.1377/hlthaff.2017.1624. PMC5989714 29733705

[R20] Grover J, 2012. A literature review of Bayes’ theorem and Bayesian belief networks (BBN). In: Strategic Economic Decision-Making: Using Bayesian Belief Networks to Solve Complex Problems. Springer, pp. 11–27.

[R21] Hébert-Dufresne L, Allard A, Garland J, Hobson EA, Zaman L, 2024. The path of complexity. npj Complex. 1 (1), 4.

[R22] Homans SW, 2007. Water, water everywhere—except where it matters? Drug Discov. Today 12 (13–14), 534–539. 17631247 10.1016/j.drudis.2007.05.004

[R23] Ireson G, 2005. Einstein and the nature of thought experiments. Sch. Sci. Rev. 86 (317), 47–53.

[R24] Jacobs DJ, Rader AJ, Kuhn LA, Thorpe MF, 2001. Protein flexibility predictions using graph theory. Proteins: Struct. Funct. Bioinform. 44 (2), 150–165. 10.1002/prot.1081 11391777

[R25] Jaynes ET, 1957a. Information theory and statistical mechanics. Phys. Rev. 106 (4), 620.

[R26] Jaynes ET, 1957b. Information theory and statistical mechanics. II. Phys. Rev. 108 (2), 171.

[R27] Jones AM, 2023. VPS26 Moonlights as an Arrestin-like Adapter for a 7-Transmembrane RGS 2 Protein in Arabidopsis thaliana. Tech. Rep, University of North Carolina, Chapel Hill, NC (United States).

[R28] Jones RD, 2024. Information transmission in G protein-coupled receptors. Int. J. Mol. Sci. 25 (3), 1621. 38338905 10.3390/ijms25031621 PMC10855935

[R29] Jones RD, Giacometti A, Jones AM, 2025a. Plumbing analog of molecular computation. arXiv preprint arXiv:2511.20339.

[R30] Jones RD, Giacometti A, Jones AM, 2025b. Thermodynamics of biological switches. arXiv preprint arXiv:2510.25359.

[R31] Jones RD, Jones AM, 2023a. Model of ligand-triggered information transmission in G-protein coupled receptor complexes. Front. Endocrinol. 14, 879. 10.3389/fendo.2023.1111594 PMC10286511 37361529

[R32] Jones RD, Jones AM, 2023b. A proposed mechanism for in vivo programming transmembrane receptors. In: Italian Workshop on Artificial Life and Evolutionary Computation. Springer, pp. 123–137.

[R33] Karshikoff A, Nilsson L, Ladenstein R, 2015. Rigidity versus flexibility: the dilemma of understanding protein thermal stability. FEBS J. 282 (20), 3899–3917. 26074325 10.1111/febs.13343

[R34] Keshelava A, Solis GP, Hersch M, Koval A, Kryuchkov M, Bergmann S, Katanaev VL, 2018. High capacity in G protein-coupled receptor signaling. Nat. Commun. 9 (1), 1–8. 29491460 10.1038/s41467-018-02868-y PMC5830429

[R35] Koenig IR, Fuchs O, Hansen G, von Mutius E, Kopp MV, 2017. What is precision medicine? Eur. Respir. J. 50 (4), 10.1183/13993003.00391-2017. 29051268

[R36] Kosorok MR, Laber EB, 2019. Precision medicine. Annu. Rev. Stat. Appl. 6, 263–286. 10.1146/annurev-statistics-030718-105251. 31073534 PMC6502478

[R37] Landauer R, 1996. The physical nature of information. Phys. Lett. A 217 (4–5), 188–193.

[R38] Latorraca NR, Masureel M, Hollingsworth SA, Heydenreich FM, Suomivuori C-M, Brinton C, Townshend RJ, Bouvier M, Kobilka BK, Dror RO, 2020. How GPCR phosphorylation patterns orchestrate arrestin-mediated signaling. Cell 183 (7), 1813–1825. 33296703 10.1016/j.cell.2020.11.014 PMC7901245

[R39] Latorraca NR, Wang JK, Bauer B, Townshend RJ, Hollingsworth SA, Olivieri JE, Xu HE, Sommer ME, Dror RO, 2018. Molecular mechanism of GPCR-mediated arrestin activation. Nature 557 (7705), 452–456. 29720655 10.1038/s41586-018-0077-3 PMC6294333

[R40] Mahoney JP, Sunahara RK, 2016. Mechanistic insights into GPCR-G protein interactions. Curr. Opin. Struct. Biol. 41, 247–254. 27871057 10.1016/j.sbi.2016.11.005 PMC5363957

[R41] Marsh JA, Teichmann SA, Forman-Kay JD, 2012. Probing the diverse landscape of protein flexibility and binding. Curr. Opin. Struct. Biol. 22 (5), 643–650. 22999889 10.1016/j.sbi.2012.08.008

[R42] Morowitz H, Smith E, 2007. Energy flow and the organization of life. Complexity 13 (1), 51–59.

[R43] Phillips R, 2021. Schrödinger’s what is life? at 75. Cell Syst. 12 (6), 465–476. 34139159 10.1016/j.cels.2021.05.013 PMC12107503

[R44] Qian H, 2007. Phosphorylation energy hypothesis: open chemical systems and their biological functions. Annu. Rev. Phys. Chem. 58, 113–142. 17059360 10.1146/annurev.physchem.58.032806.104550

[R45] Reif F, 2009. Fundamentals of Statistical and Thermal Physics. Waveland Press.

[R46] Rosing J, Slater E, 1972. The value of δG% for the hydrolysis of ATP. Biochim. et Biophys. Acta (BBA)-Bioenergetics 267 (2), 275–290. 10.1016/0005-2728(72)90116-8 4402900

[R47] Sagawa T, 2018. Second law, entropy production, and reversibility in thermodynamics of information. In: Energy Limits in Computation: A Review of Landauer’s Principle, Theory and Experiments. Springer, pp. 101–139.

[R48] Sauer MF, Sevy AM, Crowe JE Jr., Meiler J, 2020. Multi-state design of flexible proteins predicts sequences optimal for conformational change. PLoS Comput. Biol. 16 (2), e1007339. 32032348 10.1371/journal.pcbi.1007339 PMC7032724

[R49] Schrodinger R, Schrödinger E, Dinger ES, 1992. What is Life?: With Mind and Matter and Autobiographical Sketches. Cambridge University Press.

[R50] Shannon C, Weaver W, 1963. The mathematical theory of communication,(first published in 1949). Urbana University of Illinois Press.

[R51] Skiba M, Stolwijk JA, Wegener J, 2022. Label-free impedance measurements to unravel biomolecular interactions involved in G protein-coupled receptor signaling. In: Methods in Cell Biology, vol. 169, Elsevier, pp. 221–236. 35623703 10.1016/bs.mcb.2021.12.005

[R52] Stolwijk JA, Skiba M, Kade C, Bernhardt G, Buschauer A, Hübner H, Gmeiner P, Wegener J, 2019. Increasing the throughput of label-free cell assays to study the activation of G-protein-coupled receptors by using a serial agonist exposure protocol. Integr. Biology 11 (3), 99–108. 10.1093/intbio/zyz010 31083709

[R53] Teilum K, Olsen JG, Kragelund BB, 2009. Functional aspects of protein flexibility. Cell. Mol. Life Sci. 66 (14), 2231–2247. 19308324 10.1007/s00018-009-0014-6 PMC11115794

[R54] Teilum K, Olsen JG, Kragelund BB, 2011. Protein stability, flexibility and function. Biochim. et Biophys. Acta (BBA)-Proteins Proteom. 1814 (8), 969–976. 10.1016/j.bbapap.2010.11.005 21094283

[R55] Wackerhage H, Hoffmann U, Essfeld D, Leyk D, Mueller K, Zange J, 1998. Recovery of free ADP, Pi, and free energy of ATP hydrolysis in human skeletal muscle. J. Appl. Physiol. 85 (6), 2140–2145. 9843537 10.1152/jappl.1998.85.6.2140

[R56] Wegener J, Zink S, Rösen P, Galla H-J, 1999. Use of electrochemical impedance measurements to monitor β-adrenergic stimulation of bovine aortic endothelial cells. Pflügers Arch. 437, 925–934. 10370072 10.1007/s004240050864

[R57] Wirth U, Erl J, Azzam S, Höring C, Skiba M, Singh R, Hochmuth K, Keller M, Wegener J, König B, 2023. Monitoring the reversibility of GPCR signaling by combining photochromic ligands with label-free impedance analysis. Angew. Chem. 135 (21), e202215547. 10.1002/anie.202215547 36932995

[R58] Yang Z, Yang F, Zhang D, Liu Z, Lin A, Liu C, Xiao P, Yu X, Sun J-P, 2017. Phosphorylation of G protein-coupled receptors: from the barcode hypothesis to the flute model. Mol. Pharmacol. 92 (3), 201–210. 28246190 10.1124/mol.116.107839

[R59] Zhang M, Chen T, Lu X, Lan X, Chen Z, Lu S, 2024. G protein-coupled receptors (GPCRs): advances in structures, mechanisms and drug discovery. Signal Transduct. Target. Ther. 9 (1), 88. 38594257 10.1038/s41392-024-01803-6 PMC11004190

